# Supplementary material for: Causal Relationship between Aging and Anorexia Nervosa: A White-Matter-Microstructure-Mediated Mendelian Randomization Analysis
Source: Biomedicines. 2024 Aug 16;12(8):1874. doi: 10.3390/biomedicines12081874 (PMC11351342; doi:10.3390/biomedicines12081874)
Supplement: Supplementary file 1 [file biomedicines-12-01874-s001.zip › Supplementary Figure/Figure S8/Leaveoneout plot of MR analysis for TL to ubm-b-1752.pdf]

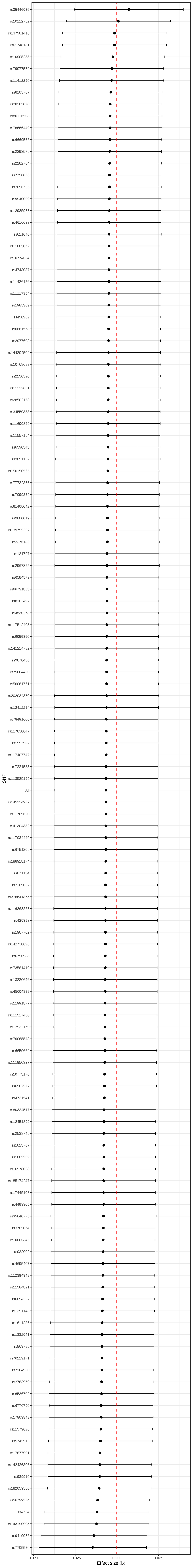
rs35446936
rs10112752
rs137901416
rs61748181
rs10905255
rs79977579
rs11412296
rs8105767
rs28363070
rs80116508
rs76666449
rs6669563
rs2293579
rs2282764
rs7790856
rs2056726
rs9940099
rs12925933
rs4616688
rs611646
rs11085072
rs10774624
rs4743037
rs11426156
rs11117354
rs1985369
rs450962
rs6881568
rs2977608
rs144204502
rs10768683
rs2230590
rs11212631
rs28502153
rs34550383
rs11699829
rs11557154
rs6590343
rs3891167
rs150150565
rs77732866
rs7099229
rs61405042
rs9600019
rs139795227
rs2276182
rs131797
rs2967355
rs6584579
rs66731853
rs8102497
rs4530278
rs117512405
rs9955360
rs141214782
rs9878436
rs75664430
rs56061761
rs202034370
rs12412214
rs78491606
rs117630647
rs1957937
rs117407747
rs7221585
rs113525195
All
rs145114957
rs11769630
rs41304832
rs117034449
rs6751209
rs188918174
rs871134
rs7209057
rs376641875
rs116863223
rs429358
rs1907702
rs142730696
rs6790988
rs73581419
rs13230646
rs45604339
rs11991877
rs111527438
rs12932179
rs76065543
rs6659669
rs111950327
rs10773176
rs6587577
rs4731541
rs80324517
rs12451892
rs2538745
rs1023767
rs1003322
rs16978028
rs185174247
rs17445108
rs4498805
rs35640778
rs3785074
rs10805346
rs932002
rs4695407
rs112394943
rs11584821
rs6054257
rs1291143
rs11611236
rs1332941
rs869785
rs76219171
rs7164950
rs2763979
rs6536702
rs6776756
rs17803849
rs11579626
rs5742915
rs17677991
rs142426306
rs939916
rs182059586
rs56799554
rs4724
rs143190905
rs9419958
rs7705526
SNP
-0.050
-0.025
0.000
0.025
Effect size (b)
